# A Risk Model Incorporating the Novel Inflammatory Biomarker CD64 for Predicting Bloodstream Infection in Suspected Cases

**DOI:** 10.3390/antibiotics15030322

**Published:** 2026-03-23

**Authors:** Teng Xu, Yu Zhou, Bei Wang, Li Wang, Yinglu Wan, Shi Wu, Haihui Huang

**Affiliations:** 1 Institute of Antibiotics, Huashan Hospital, Fudan University, Shanghai 200040, China; txu20@fudan.edu.cn (T.X.); 22211220091@fudan.edu.cn (Y.Z.); wanglishiny@163.com (L.W.); wan_yinglu@163.com (Y.W.); 2 Key Laboratory of Clinical Pharmacology of Antibiotics, National Health and Family Planning Commission, Shanghai 200040, China; 3 Department of Laboratory Medicine, Huashan Hospital, Fudan University, Shanghai 200040, China; wa0261008@163.com

**Keywords:** bloodstream infection, CD64, diagnostic performance, nomogram, serum inflammatory marker

## Abstract

**Background/Objectives**: Bloodstream infection (BSI) is a significant cause of mortality. The availability of a convenient tool for predicting the risk of BSI at the early stage would be beneficial for clinicians, allowing them to improve the outcomes of BSI and avoid antibiotic overuse. **Methods**: A multivariate prediction model was constructed based on conventional laboratory test results and novel serum inflammatory markers in a cohort of patients with suspected BSI over a one-year period using least absolute shrinkage and selection operator (LASSO) and logistic regression. **Results**: BSI was confirmed in 99 (32.0%) of the 309 enrolled patients. Five readily available markers were identified as independent predictors: the presence of local infection, platelet count, and C-reactive protein, procalcitonin (PCT), and CD64 levels. A nomogram based on these five variables achieved an area under the receiver operating characteristic curve of 0.85 in predicting the risk of BSI. The nomogram was superior to PCT alone in terms of the net clinical benefits obtained in a rather wide range of threshold probabilities. **Conclusions**: The simple five-variable nomogram developed in this study is useful for timely prediction of individuals at high risk of BSI. It may be used in clinical practice to facilitate timely decision-making on antimicrobial treatment and avoid inappropriate overuse of antibiotics.

## 1. Introduction

Bloodstream infection (BSI) continues to be a frequent and significant cause of fatality [1]. Data from a nationwide Finnish cohort (2004–2018) show that the annual incidence of BSI almost doubled, rising from 150 to 309 cases per 100,000 population, while one-month all-cause mortality rose in parallel from 20 to 39 deaths per 100,000 [2]. A recent multi-center study covering six German tertiary-care university hospitals found the in-hospital mortality to be 23.8%, with six-month mortality reaching 41.6% [3]. Mortality is even higher among neonates, immunocompromised hosts, and the critically ill [4,5,6].

Early detection is critical for better outcomes of BSI. Blood culture is the well-recognized gold standard for diagnosing BSI. However, the bacterial load in blood is typically lower than 100 colony-forming units (CFUs) per milliliter [7,8], leading to a low (5–10%) positivity rate for blood cultures. Furthermore, the time-consuming blood culture process usually results in a 48–72 h delay to reach an evidence-based treatment decision [9]. The next-generation sequencing (NGS) technique has improved the detection rate of BSI. However, the interpretation of NGS results depends on expertise and careful analysis, and the method’s high cost prevents its use in routine diagnostics [10]. Some readily accessible inflammatory markers, such as C-reactive protein (CRP), procalcitonin (PCT), and interleukin-6 (IL-6), can be used to identify BSIs. These biomarkers are not specific for BSIs because elevated levels of these biomarkers are also observed in local infections without bacteremia or even in non-infectious diseases [11,12,13]. Therefore, it is reasonable to develop a better tool for the early detection of BSIs by integrating multiple novel promising biomarkers with biochemical variables.

CD64 is a high-affinity receptor for the Fcγ segment of IgG. The serum level of CD64 is positively correlated with the severity of infection [14]. CD64 demonstrates high diagnostic accuracy, with meta-analyses reporting approximately 87% sensitivity and specificity for sepsis detection [15]. CD64 can be effectively used to distinguish bacterial from viral infections [16]. CD64’s superior kinetic profile, diagnostic precision, and capacity for therapeutic monitoring position it as a valuable marker in infection management. Interleukin-17A (IL-17A) is a pro-inflammatory cytokine produced by Th17 cells. Interleukin-36 (IL-36) consists of three agonists (IL-36α, IL-36β, and IL-36γ) and one antagonist (IL-36Ra). IL-17A and IL-36 perform well in the early diagnosis of BSIs and sepsis, respectively, evidenced by their area under the receiver operating characteristic curve (AUC) values [17,18]. However, these emerging biomarkers have primarily been evaluated in localized infectious diseases. More clinical research data are still required to support their use in BSI populations.

In the present study, we developed a simple scoring system by integrating promising novel biomarkers with routine clinical variables to facilitate the early identification of BSI within 24 h after admission.

## 2. Results

Of the 309 patients who underwent blood culture testing, 99 (32.0%) fulfilled the criteria for microbiologically confirmed BSI. Compared to non-BSI patients, those with BSI exhibited significantly higher white blood cell counts (WBCs), neutrophil percentages, total bilirubin, direct bilirubin, serum urea nitrogen, and serum creatinine, as well as lower platelet counts (PLTs), hemoglobin, and serum albumin. A history of trauma or surgery, catheter use, and the presence of an implantable device were also more frequently observed in BSI patients (Table 1). Furthermore, local infections including respiratory tract infection, urinary tract infection, abdominal infection, skin and soft tissue infection, and central nervous system infection were significantly more common in the BSI group than in the non-BSI group (Supplementary Figure S1).

### 2.1. Performance of Serum Inflammatory Markers in Identifying BSI

**Figure 1:**
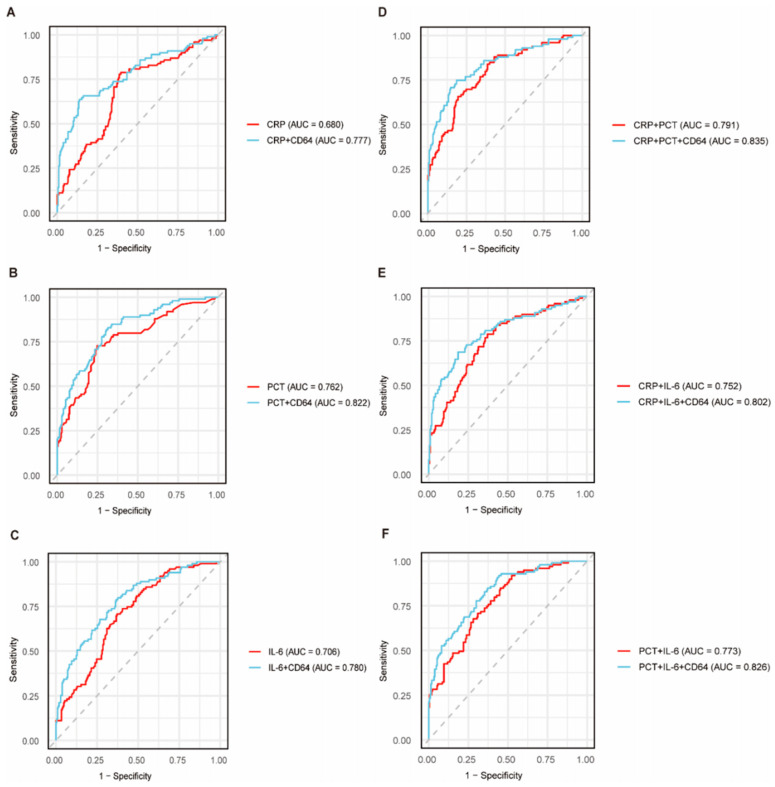
ROC curves of conventional biomarkers alone or in combination with CD64 for diagnosing bloodstream infection. (**A**) CRP alone and in combination with CD64; (**B**) PCT alone and in combination with CD64; (**C**) IL-6 alone and in combination with CD64; (**D**) CRP in combination with PCT and CD64; (**E**) CRP in combination with IL-6 and CD64; (**F**) PCT in combination with IL-6 and CD64.CRP, C-reactive protein; AUC, area under the curve; PCT, procalcitonin; ROC, receiver operating characteristic.

The levels of inflammatory markers at the time the blood culture samples were drawn are depicted in Supplementary Figure S2. BSI was associated with significantly higher CRP (60.5 vs. 30.4 mg/L, *p* < 0.001), PCT (0.44 vs. 0.14 ng/mL, *p* < 0.001), IL-6 (85.48 vs. 29.29 pg/mL, *p* < 0.001), IL-7A (2.95 vs. 1.75 pg/mL, *p* < 0.001), CD64 (3.63 vs. 1.20 pg/mL, *p* < 0.001), and IL-36γ (572.48 vs. 236.73 pg/mL, *p* < 0.001) levels as compared with those for the febrile patients without BSI. Among the emerging biomarkers, CD64 resulted in a greater AUC (0.746, 95% CI: 0.686–0.707) than IL-17A (0.715, 95% CI: 0.654–0.777) and IL-36 (0.661, 95% CI: 0.599–0.724) but a lesser AUC than PCT (0.762, 95% CI: 0.703–0.821) in diagnosing BSI in the ROC analysis (Supplementary Figure S3). Combination with CD64 significantly enhanced the diagnostic performance of the conventional biomarkers. The PCT-CD64 combination in particular increased the AUC to 0.822 (95% CI: 0.772–0.871), which was higher than that for any other combination (Figure 1).

### 2.2. Development and Utility of the Prediction Model

All the continuous variables, such as CRP, WBC, and blood urea nitrogen, showed a monotonic association with BSI. These variables and other binary variables were entered directly into a LASSO regression model without variable transformation (Supplementary Figure S4) in the training set. Supplementary Figure S4A shows the dynamic shrinkage process of each variable’s coefficient as the regularization parameter λ increased. At the optimal λ value, determined by minimizing the mean squared error, seven robust predictors were retained. Multivariate analysis further narrowed the predictive factors down to a combination of local infection, PLT, and CRP, PCT, and CD64 levels (Table 2).

**Figure 2:**
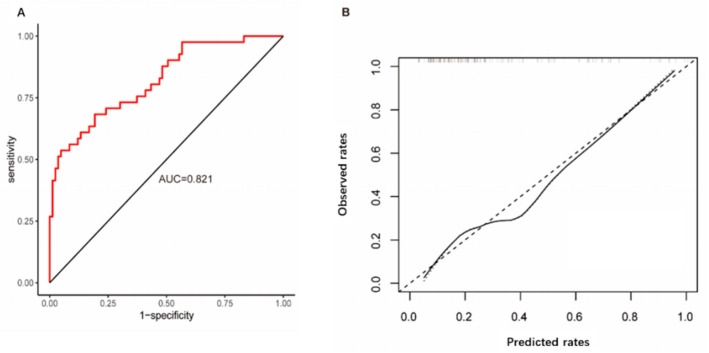
Calibration and discrimination of the predictive model for bloodstream infection in patients with fever of unknown origin. (**A**) Discrimination of BSI status by the predictive model using the area under the receiver operating characteristic curve. (**B**) External calibration curves in the validation cohort. AUC, area under the receiver operating characteristic curve.

A logistic regression model incorporating these five predictors demonstrated strong discriminatory power, with a C-statistic of 0.90, and good calibration, as indicated by a nonsignificant Hosmer–Lemeshow test (χ^2^ = 5.21; *p* > 0.05) during internal validation. Bootstrap resampling revealed no substantial overfitting, yielding an optimism-corrected C-statistic of 0.88 and a calibration slope of 0.90. When applied to the external validation set, the model exhibited satisfactory performance, achieving an area under the ROC curve of 0.821 (95% CI: 0.741–0.900). Furthermore, the predicted incidence of BSI based on the risk score closely aligned with the observed incidence, as illustrated in Figure 2.

**Figure 3:**
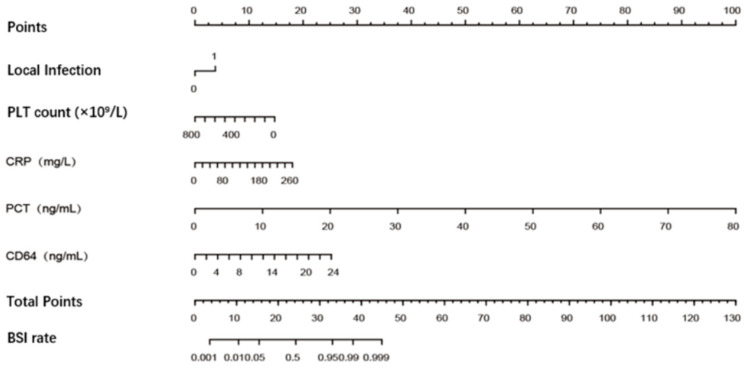
Nomogram for predicting bloodstream infections. PLT, platelet; CRP, C-reactive protein; PCT, procalcitonin; BSI, bloodstream infection.

**Figure 4:**
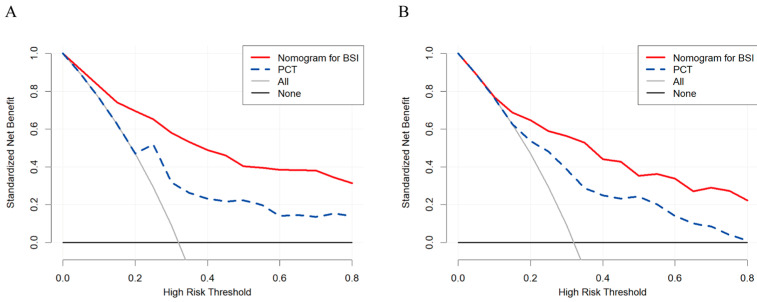
The DCA-defined clinical utility of the nomogram and PCT in predicting the risk of BSI in the training set (**A**) and the validation set (**B**). BSI, bloodstream infection; PCT, procalcitonin; DCA, decision curve analysis.

Finally, our predictive model was visualized as a nomogram (Figure 3). The nomogram is promising for predicting BSI in clinical practice because of the wide and practical range of threshold probabilities in both the training and validation sets. The five-variable model developed in this study is superior to the well-recognized biomarker PCT alone because the newly developed tool achieves more clinical net benefits in a rather wide range of threshold probabilities (Figure 4).

## 3. Discussion

We developed a novel and practical diagnostic tool for the early prediction of BSI risk in suspected cases. The tool, presented as a nomogram, incorporates five variables: the presence of local infection, PLT, and CRP, PCT, and CD64 levels. These parameters are routinely documented and electronically retrievable in tertiary-care centers, enabling real-time estimation of BSI risk at the bedside. The scoring tool demonstrated robust discrimination and calibration upon both internal and external validation.

Conventional inflammatory biomarkers such as procalcitonin (PCT) and C-reactive protein (CRP) exhibit limited accuracy when used alone for the identification of bloodstream infection (BSI) [19,20,21]. Although PCT demonstrates greater predictive value than CRP, its discriminative performance, as reflected by the area under the curve (AUC), varies considerably (ranging from 0.70 to 0.90) across different clinical settings and patient populations [22,23,24]. In our cohort of patients with suspected BSI, a PCT level of >0.25 ng/mL within 24 h of admission yielded only moderate diagnostic accuracy (AUC = 0.76; 95% CI: 0.70–0.82) with suboptimal sensitivity (0.73), albeit with higher specificity (0.75) compared to CRP (0.61). Il-6 had only moderate diagnostic value (combined AUC 0.80, 95% CI: 0.76–0.83) for sepsis in adults, with significantly lower sensitivity as compared to PCT and CRP [25], which is consistent with our findings. One possible reason for this is the short half-life of IL-6 (1–4 h), which might lead to false negatives if detected after the peak [26]. Prior antibiotic exposure was not an exclusion criterion in this study. Such exposure may attenuate biomarker levels by reducing the bacterial load and blunting the inflammatory response, potentially leading to an underestimation of BSI risk. While this confounding effect may influence model performance, its inclusion enhances the model’s applicability to real-world settings. Future studies could collect detailed antibiotic data to further refine the model.

Integrated diagnostic models, particularly those leveraging machine learning (ML) approaches, can enhance the early detection of BSI, thereby facilitating the timely initiation of empirical antimicrobial therapy [27,28,29]. On the contrary, the development and deployment of these models are fraught with significant challenges. The “black-box” nature of complex ML algorithms often obscures the reasoning behind predictions, raising concerns about interpretability and hindering clinical trust and adoption [30]. The integration of novel biomarkers or NGS data into these models, while potentially improving performance, also introduces preanalytical and analytical variabilities, such as batch effects and pathogen DNA load biases, which can compromise robustness [31]. Additionally, the machine learning approach requires a comprehensive electronic database. All parameters related to patient characteristics must be available for the algorithm to work. Therefore, it is not readily applicable to most healthcare settings. In the present study, a simple and practical nomogram was designed for bedside use. The predictors are well defined, easily measured, and readily available.

The scoring tool performed well in identifying BSI in suspected cases (AUC, 0.85). The innovative value of our model is further highlighted by the specific contribution of CD64. Our data show that CD64 had a superior diagnostic odds ratio when compared to other novel inflammatory biomarkers, and, more importantly, its addition to the PCT-CD64 combination yielded the highest AUC (0.822). This suggests that CD64 captures a distinct aspect of the host immune response to bacteremia that complements PCT. It should be noted that serum CD64 levels were measured using an enzyme-linked immunosorbent assay (ELISA) due to its feasibility for batch testing and compatibility with stored serum samples. Although flow cytometry is the standard method for assessing neutrophil CD64 expression, soluble CD64 detected via ELISA and neutrophil CD64 detected via flow cytometry have similar AUCs and specificity in inflammatory diseases, with even superior sensitivity [32]. Numerous studies have demonstrated that CD64 is more specific than CRP and PCT in diagnosing sepsis [15,33]. CD64 is primarily expressed on neutrophils, monocytes, and macrophages, and its expression is significantly upregulated during bacterial infections, especially sepsis. In contrast, the elevation of CD64 is less pronounced in viral infections or non-infectious inflammatory diseases because neutrophils are less involved in these pathophysiological processes [34]. The newly developed model combines CD64 with PCT and CRP, which helps address the low diagnostic specificity of conventional inflammatory markers for diagnosing BSI. It is interesting to note that the PLT was selected as one of the predictors. Many studies indicate that the PLT can serve as a prognostic factor for sepsis. Persistent thrombocytopenia (especially < 50 × 10^9^/L) suggests condition deterioration and is associated with organ failure and increased mortality [35]. Zhou P also reported thrombocytopenia in populations with BSI caused by *Escherichia coli* and *Klebsiella pneumoniae* [36]. In the present study, more than 50% of the patients with BSI had thrombocytopenia (PLT < 100 × 10^9^/L).

Early antimicrobial therapy can improve patient outcomes for BSI. On the contrary, unnecessary antimicrobial therapy increases the risk of adverse drug reactions and secondary infections and promotes the spread of antibiotic-resistant bacteria [37]. The clinical importance of this research is best illustrated by its potential impact on antimicrobial stewardship. The proposed nomogram has the potential to help clinicians assess the possibility of BSI before knowing the results of blood culture and decide whether anti-infective treatment needs to be initiated immediately. In our center, if a PCT of 0.5 ng/mL (indicating a high risk of sepsis) was used as the criterion to initiate antimicrobial therapy for BSI, 51 patients (52%) with BSI would not receive timely antimicrobial treatment, and 37 non-BSI patients (18%) would receive unnecessary antibiotic prescriptions. However, if the newly developed nomogram—using a cut-off of ≥25 (a round number that is easy to remember and apply, representing a balanced trade-off between false-positive and false-negative rates)—was used to guide antibiotic therapy for BSI, only 25 non-BSI patients (25%) would not receive timely antimicrobial treatment, while only 30 (14%) non-BSI patients would receive unnecessary antibiotic prescriptions. Crucially, the nomogram demonstrates significant potential as a bedside decision-support tool for use within 24 h of admission, enabling timely treatment decisions before blood culture results are available.

There are some limitations to this study. The nature of this single-center study may have introduced selection bias and center effects; further validation in independent external cohorts is warranted. Additionally, the BSI cases in this study were primarily caused by Gram-negative bacteria (72.6%). Further studies are needed to validate the performance of this nomogram for BSIs caused by Gram-positive bacteria or fungal pathogens.

## 4. Materials and Methods

### 4.1. Study Design

Patients with suspected BSI who underwent blood culture testing at Huashan Hospital, Fudan University (a 3142-bed tertiary teaching hospital in Shanghai, China), between May 2024 and January 2025, were enrolled in this study.

This study received ethical approval from the Institutional Review Board of Huashan Hospital, Fudan University (approval No. 2024-607). All participants provided written informed consent prior to their enrollment. The conduct and reporting of this study adhered to the TRIPOD guidelines for transparent reporting of multivariable prediction models [38].

### 4.2. Study Population

All enrolled patients were adults (aged 18 years or older) satisfying the following criteria: (1) fever, chills, and other signs of infection; (2) suspected BSI and blood samples sent for blood culture testing; and (3) CRP and PCT tested on the same day as the blood culture. Patients were excluded in case of (1) prior enrollment within the past month; (2) incomplete clinical data; or (3) known human immunodeficiency virus infection. Patients were withdrawn when (1) blood samples were contaminated or highly suspected of contamination, evidenced by a positive result after 48 h, or the growth of common contaminants such as coagulase-negative Staphylococcus or Bacillus in a single culture, or (2) informed consent was withdrawn.

BSI was defined by positive blood cultures (excluding Corynebacterium species) in a patient with systemic signs of infection. However, multiple positive cultures for common commensals (e.g., coagulase-negative Staphylococcus) were considered true BSI if the second culture occurred on the same or subsequent day with a consistent clinical context, such as the presence of systemic signs of infection, risk factors (e.g., indwelling catheters), and no alternative explanation for the clinical presentation [39].

### 4.3. Data Collection

Clinical and laboratory data were collected, including patient demographics, symptoms and signs, underlying diseases, laboratory results, and discharge diagnoses. Documentation was made of any invasive procedures performed. Corticosteroid use was defined as the administration of prednisone at an equivalent dosage of over 15 mg per day. Chemotherapy was defined as treatment with cytotoxic anti-tumor drugs with curative or palliative intent. Blood culture results were rigorously assessed.

### 4.4. Laboratory Tests

For all participants, a 5 mL blood sample was collected on the same day as the blood culture sample to test for the biomarkers IL-6, IL-17A, CD64, and IL-36γ. The blood samples were centrifuged at 3000 rpm for 10 min to separate serum. The prepared serum samples were aliquoted into EP tubes and stored at −80 °C until the assay to reduce protein degradation and ensure the reliability of the test results. Serum levels of IL-6, IL-17A, CD64, and IL-36γ were measured using an enzyme-linked immunosorbent assay (ELISA) (IL-6 and IL-17A kits, Multi Sciences Biotech, Hangzhou, China; CD64 and IL-36γ kits, CUSABIO, Wuhan, China).

### 4.5. Statistical Analysis

The continuous variables were evaluated by using the Kolmogorov–Smirnov test to verify the normality of their distribution, then compared between groups using Student’s *t*-test or the Mann–Whitney U-test. The categorical variables were compared between groups using Pearson’s Chi-square test or Fisher’s exact test. The difference was considered statistically significant when the two-sided *p* value was <0.05. Imputation for missing variables was considered if missing values comprised less than 20%, using a previously described method [40]. Otherwise, the variable was removed from the analysis.

Overall, 60% of the samples were designated as the model training set, and the remaining 40% of the samples were designated as the model validation set. The training set consisted of 185 patients with 59 (31.9%) cases of BSI. The validation set consisted of 124 patients with 40 (32.2%) cases of BSI. To mitigate potential issues of multicollinearity and overfitting, the least absolute shrinkage and selection operator (LASSO) regression method was employed [40].

Model discrimination was assessed using the receiver operating characteristic (ROC) curve and the corresponding area under the curve (AUC). Calibration was evaluated with the Hosmer–Lemeshow test and visualized through calibration plots to examine goodness-of-fit. After internal validation via bootstrap resampling (1000 repetitions), the regression model was further subjected to internal split-sample validation using the validation set. Decision curve analysis (DCA) was employed to quantify the clinical utility of the model across various risk thresholds [41]. An optimal predictor of BSI would demonstrate superior net clinical benefit over a broad range of threshold probabilities. The final model was presented as a nomogram, constructed according to previously established methods [42]. All statistical analyses were conducted using R software (version 4.0.2; R Foundation, Vienna, Austria) and SPSS (version 25.0; IBM, Armonk, NY, USA).

## 5. Conclusions

A simple nomogram was developed by combining five variables—the presence of local infection, PLT, and CRP, PCT, and CD64 levels—in a cohort of 309 patients. The proposed tool is helpful for clinicians to identify patients at a high risk of BSI. Moreover, the clinical use of this tool may facilitate timely antimicrobial treatment decision and avoid inappropriate overuse of antibiotics.

## Figures and Tables

**Table 1:** Demographics and clinical characteristics of patients.

Characteristic	Total	Bloodstream Infection		*p* Value
		Yes	No	
Number of patients	309	99	210	NA
Demographics				
Age (years)	61 (49–72)	63 (53–74)	59 (47–72)	0.131
Male sex	106 (34.0)	35 (35.0)	71 (71.0)	0.890
Comorbidity				
Diabetes mellitus	76 (25.0)	26 (26.0)	50 (24.0)	0.745
Heart failure	8 (3.0)	2 (2.0)	6 (3.0)	1.000
Liver cirrhosis	54 (17.0)	23 (23.0)	31 (15.0)	0.095
Chronic kidney disease	23 (7.0)	9 (9.0)	14 (7.0)	0.599
Collective tissue disease	18 (6.0)	5 (5.0)	13 (6.0)	0.889
Solid tumor	52 (17.0)	22 (22.0)	30 (14.0)	0.115
Hematological malignancy	14 (5.0)	5 (5.0)	9 (4.0)	0.774
Local infection	151 (49.0)	27 (27.0)	124 (59.0)	<0.01
Other underlying condition				
Corticosteroid use ^a^	52 (17.0)	21 (21.0)	31 (15.0)	0.211
Immunosuppressants	20 (6.0)	12 (12.0)	8 (4.0)	0.012
Chemotherapy ^a^	13 (4.0)	5 (5.0)	8 (4.0)	0.012
Invasive procedure ^a^	75 (24.0)	38 (38.0)	37 (18.0)	0.006
Catheter use	141 (45.6)	81 (81.8)	60 (28.6)	0.0013
Laboratory analysis				
Hemoglobin (g/L)	112 (92, 131)	94 (80, 111)	119 (103, 134)	<0.001
White blood cell count (10^9^/L)	8.03 (5.66, 11.51)	10.16 (6.01, 13.56)	7.66 (5.6, 10.83)	0.034
Platelet count (10^9^/L)	178 (108, 260)	141 (63, 200)	201 (133, 290)	<0.001
Alanine aminotransferase (U/L)	30 (16, 67)	30.5 (15, 59.25)	29 (17, 97.5)	0.337
Aspartate aminotransferase (U/L)	33 (20, 68)	38 (20, 83.5)	29.5 (20, 58.25)	0.158
Total bilirubin (μmol/L)	13.7 (9, 29.3)	20.2 (11.45, 49)	11.5 (8.35, 21.4)	<0.001
Direct bilirubin (μmol/L)	3.4 (1.4, 7.9)	5.2 (1.3, 17.75)	2.8 (1.4, 5.88)	0.006
Globin (g/L)	35 (30, 38)	33 (28.5, 36)	35 (31, 40)	<0.001
Blood urea nitrogen (mmol/L)	6 (4.5, 8.9)	8.1 (5.45, 12.6)	5.5 (4.3, 7.6)	<0.001
Serum creatinine (μmol/L)	61 (47, 83)	68 (52.5, 126)	59 (46, 75.5)	0.002

Data are presented as *n* (%) or median (interquartile range) unless otherwise specified. NA, not applicable. ^a^ Within 30 days before admission.

**Table 2:** Multivariate logistic regression model and scoring system for predicting the risk of BSI in adult patients.

Variable	β-Coefficient	OR	95% CI	*p* Value
Local infection	1.295	3.650	1.447–9.208	0.006
Platelet count	−0.005	0.995	0.991–0.999	0.013
C-reactive protein	0.023	1.024	1.013–1.035	0.000
Procalcitonin	0.409	1.505	1.014–2.233	0.042
CD64	0.344	1.411	1.166–1.708	0.000

## Data Availability

The datasets are not publicly available due to privacy or ethical restrictions. The data that support the findings of this study are available from the corresponding author upon reasonable request.

## Supplementary Materials

The following supporting information can be downloaded at https://www.mdpi.com/article/10.3390/antibiotics15030322/s1. Figure S1: Distribution of primary site of infection in patients with or without bloodstream infection; Figure S2: Serum levels of inflammatory markers at the time blood culture samples were drawn from patients with BSI; Figure S3: The areas under the receiver operating characteristic curves and the corresponding 95% CIs of relevant biomarkers for diagnosing bloodstream infections; Figure S4: Variable selection using the least absolute shrinkage and selection operator (LASSO) binary logistic regression model; Table S1: Demographics and clinical characteristics of patients in the training and validation sets.

## Abbreviations

The following abbreviations are used in this manuscript:

BSI	Bloodstream infection
CFUs	Colony-forming units
NGS	Next-generation sequencing
CRP	C-reactive protein
PCT	Procalcitonin
AUC	Area under the receiver operating characteristic curve
ELISA	Enzyme-linked immunosorbent assay
LASSO	Least absolute shrinkage and selection operator
ROC	Receiver operating characteristic
DCA	Decision curve analysis
WBC	White blood cell count
PLT	Platelet count

## References

[1] Timsit J.F., Ruppé E., Barbier F., Tabah A., Bassetti M. (2020). Bloodstream infections in critically ill patients: An expert statement. Intensive Care Med..

[2] Kontula K.S.K., Skogberg K., Ollgren J., Järvinen A., Lyytikäinen O. (2021). Population-Based Study of Bloodstream Infection Incidence and Mortality Rates, Finland, 2004–2018. Emerg. Infect. Dis..

[3] Tacconelli E., Göpel S., Gladstone B.P., Eisenbeis S., Hölzl F., Buhl M., Górska A., Cattaneo C., Mischnik A., Rieg S. (2022). Development and validation of BLOOMY prediction scores for 14-day and 6-month mortality in hospitalised adults with bloodstream infections: A multicentre, prospective, cohort study. Lancet Infect. Dis..

[4] Jennings M.R., Elhaissouni N., Colantuoni E., Prochaska E.C., Johnson J., Xiao S., Clark R.H., Greenberg R.G., Benjamin D.K., Milstone A.M. (2025). Epidemiology and Mortality of Invasive Staphylococcus aureus Infections in Hospitalized Infants. JAMA Pediatr..

[5] Adrie C., Garrouste-Orgeas M., Ibn Essaied W., Schwebel C., Darmon M., Mourvillier B., Ruckly S., Dumenil A.-S., Kallel H., Argaud L. (2017). Attributable mortality of ICU-acquired bloodstream infections: Impact of the source, causative micro-organism, resistance profile and antimicrobial therapy. J. Infect..

[6] Kern W.V., Rieg S. (2020). Burden of bacterial bloodstream infection-a brief update on epidemiology and significance of multidrug-resistant pathogens. Clin. Microbiol. Infect..

[7] Filbrun A.B., Richardson J.C., Khanal P.C., Tzeng Y.L., Dickson R.M. (2022). Rapid, label-free antibiotic susceptibility determined directly from positive blood culture. Cytom. A.

[8] Wain J., Diep T.S., Ho V.A., Walsh A.M., Hoa N.T.T., Parry C.M., White N.J. (1998). Quantitation of bacteria in blood of typhoid fever patients and relationship between counts and clinical features, transmissibility, and antibiotic resistance. J. Clin. Microbiol..

[9] Biondi E.A., Mischler M., Jerardi K.E., Statile A.M., French J., Evans R., Lee V., Chen C., Asche C., Ren J. (2014). Pediatric Research in Inpatient Settings (PRIS) Network. Blood culture time to positivity in febrile infants with bacteremia. JAMA Pediatr..

[10] Wang Y., Lindsley K., Bleak T.C., Jiudice S., Uyei J., Gu Y., Wang Y., Timbrook T.T., Balada-Llasat J.-M. (2025). Performance of molecular tests for diagnosis of bloodstream infections in the clinical setting: A systematic literature review and meta-analysis. Clin. Microbiol. Infect..

[11] Harikrishna J., Mohan A., Kalyana Chakravarthi D.P., Chaudhury A., Kumar B.S., Sarma K.V.S. (2020). Serum procalcitonin as a biomarker of bloodstream infection & focal bacterial infection in febrile patients. Indian J. Med. Res..

[12] Kasperska-Zajac A., Grzanka A., Machura E., Mazur B., Misiolek M., Czecior E., Kasperski J., Jochem J. (2013). Analysis of procalcitonin and CRP concentrations in serum of patients with chronic spontaneous urticaria. Inflamm. Res..

[13] Mućka S., Jakubiak G.K., Pawlas N. (2025). Procalcitonin: Infection or Maybe Something More? Noninfectious Causes of Increased Serum Procalcitonin Concentration: Updated Knowledge. Life.

[14] Cid J., Aguinaco R., Sánchez R., García-Pardo G., Llorente A. (2010). Neutrophil CD64 expression as marker of bacterial infection: A systematic review and meta-analysis. J. Infect..

[15] Lan H.M., Wu C.C., Liu S.H., Li C.-H., Tu Y.-K., Chen K.-F. (2025). Comparison of the diagnostic accuracies of various biomarkers and scoring systems for sepsis: A systematic review and Bayesian diagnostic test accuracy network meta-analysis. J. Crit. Care.

[16] Yu L., Cen P., Zhang L., Ke J., Xu X., Ding J., Jin J., Leng J., Yu Y. (2023). Neutrophil CD64 index as a good biomarker for early diagnosis of bacterial infection in pregnant women during the flu season. Influenza Other Respir. Viruses.

[17] Frimpong A., Owusu E.D.A., Amponsah J.A., Obeng-Aboagye E., van der Puije W., Frempong A.F., Kusi K.A., Ofori M.F. (2022). Cytokines as Potential Biomarkers for Differential Diagnosis of Sepsis and Other Non-Septic Disease Conditions. Front. Cell Infect. Microbiol..

[18] Wang H., Wang M., Chen J., Hou H., Guo Z., Yang H., Tang H., Chen B. (2023). Interleukin-36 is overexpressed in human sepsis and IL-36 receptor deletion aggravates lung injury and mortality through epithelial cells and fibroblasts in experimental murine sepsis. Crit. Care.

[19] Reintam Blaser A., Starkopf J., Björck M., Forbes A., Kase K., Kiisk E., Laisaar K.-T., Mihnovits V., Murruste M., Mändul M. (2023). Diagnostic accuracy of biomarkers to detect acute mesenteric ischaemia in adult patients: A systematic review and meta-analysis. World J. Emerg. Surg..

[20] Hoeboer S.H., van der Geest P.J., Nieboer D., Groeneveld A.B. (2015). The diagnostic accuracy of procalcitonin for bacteraemia: A systematic review and meta-analysis. Clin. Microbiol. Infect..

[21] Lee C.C., Hong M.Y., Lee N.Y., Chen P.L., Chang C.M., Ko W.C. (2012). Pitfalls in using serum C-reactive protein to predict bacteremia in febrile adults in the ED. Am. J. Emerg. Med..

[22] El Haddad H., Chaftari A.M., Hachem R., Chaftari P., Raad I.I. (2018). Biomarkers of Sepsis and Bloodstream Infections: The Role of Procalcitonin and Proadrenomedullin With Emphasis in Patients With Cancer. Clin. Infect. Dis..

[23] Lin J.C., Chen Z.H., Chen X.D. (2020). Elevated serum procalcitonin predicts Gram-negative bloodstream infections in patients with burns. Burns.

[24] Sun Q., Lin Q., Lv Y., Tian Z., Yan Q., Yu Y., Fu X., Yao H., Sun F., Xia Y. (2025). Predictive value of serum procalcitonin level for the diagnosis of bloodstream infections in hematological patients. BMC Infect. Dis..

[25] Han Z., Li J., Yi X., Zhang T., Liao D., You J., Ai J. (2024). Diagnostic accuracy of interleukin-6 in multiple diseases: An umbrella review of meta-analyses. Heliyon.

[26] Guba S.C., Sartor C.I., Gottschalk L.R., Jing Y.H., Mulligan T., Emerson S.G. (1992). Bone marrow stromal fibroblasts secrete interleukin-6 and granulocyte-macrophage colony-stimulating factor in the absence of inflammatory stimulation: Demonstration by serum-free bioassay, enzyme-linked immunosorbent assay, and reverse transcriptase polymerase chain reaction. Blood.

[27] Malic L., Zhang P.G.Y., Plant P.J., Clime L., Nassif C., Da Fonte D., Haney E.E., Moon B.-U., Sit V.M.-S., Brassard D. (2025). A machine learning and centrifugal microfluidics platform for bedside prediction of sepsis. Nat. Commun..

[28] Villani Júnior A., Freire M.P., Lazar Neto F., De Padua Covas Lage L.A., Oliveira M.S., Abdala E., Nunes F.L.S., Levin A.S.S. (2025). Prediction of bacterial and fungal bloodstream infections using machine learning in patients undergoing chemotherapy. Eur. J. Cancer.

[29] Yesil M.R., Talli I., Pelloso M., Cosma C., Pangrazzi E., Plebani M., Ustundag Y., Padoan A. (2025). Impact of analytical bias on machine learning models for sepsis prediction using laboratory data. Clin. Chem. Lab. Med..

[30] Wiens J., Shenoy E.S. (2018). Machine Learning for Healthcare: On the Verge of a Major Shift in Healthcare Epidemiology. Clin. Infect. Dis..

[31] Su M., Satola S.W., Read T.D. (2019). Genome-Based Prediction of Bacterial Antibiotic Resistance. J. Clin. Microbiol..

[32] Minar P., Jackson K., Tsai Y.T., Sucharew H., Rosen M.J., A Denson L. (2017). Validation of Neutrophil CD64 Blood Biomarkers to Detect Mucosal Inflammation in Pediatric Crohn’s Disease. Inflamm. Bowel Dis..

[33] Nuutila J. (2010). The novel applications of the quantitative analysis of neutrophil cell surface FcgammaRI (CD64) to the diagnosis of infectious and inflammatory diseases. Curr. Opin. Infect. Dis..

[34] Dimoula A., Pradier O., Kassengera Z., Dalcomune D., Turkan H., Vincent J.L. (2014). Serial determinations of neutrophil CD64 expression for the diagnosis and monitoring of sepsis in critically ill patients. Clin. Infect. Dis..

[35] Xu Y., Jin X., Shao X., Zheng F., Zhou H. (2018). Valuable prognostic indicators for severe burn sepsis with inhalation lesion: Age, platelet count, and procalcitonin. Burn. Trauma.

[36] Li S., Yu S., Qin J., Peng M., Qian J., Zhou P. (2022). Prognostic value of platelet count-related ratios on admission in patients with pyogenic liver abscess. BMC Infect. Dis..

[37] Anderer S. (2025). Shorter Antibiotic Course Effective for Bloodstream Infections. JAMA.

[38] Collins G.S., Reitsma J.B., Altman D.G., Moons K.G. (2015). Transparent Reporting of a multivariable prediction model for Individual Prognosis or Diagnosis (TRIPOD): The TRIPOD statement. Ann. Intern. Med..

[39] Xu T., Wu S., Li J., Wang L., Huang H. (2022). Development of a risk prediction model for bloodstream infection in patients with fever of unknown origin. J. Transl. Med..

[40] Zipursky A.R., Yoon E.W., Emberley J., Bertelle V., Kanungo J., Lee S.K., Shah P.S. (2019). Canadian Neonatal Network Investigators. Central Line-Associated Blood Stream Infections and Non-Central Line-Associated Blood Stream Infections Surveillance in Canadian Tertiary Care Neonatal Intensive Care Units. J. Pediatr..

[41] Van Calster B., Wynants L., Verbeek J.F.M., Verbakel J.Y., Christodoulou E., Vickers A.J., Roobol M.J., Steyerberg E.W. (2018). Reporting and Interpreting Decision Curve Analysis: A Guide for Investigators. Eur. Urol..

[42] Morris C.K., Myers J., Froelicher V.F., Kawaguchi T., Ueshima K., Hideg A. (1993). Nomogram based on metabolic equivalents and age for assessing aerobic exercise capacity in men. J. Am. Coll. Cardiol..

