# Supplementary material for: A Risk Model Incorporating the Novel Inflammatory Biomarker CD64 for Predicting Bloodstream Infection in Suspected Cases
Source: Antibiotics (Basel). 2026 Mar 23;15(3):322. doi: 10.3390/antibiotics15030322 (PMC13023907; doi:10.3390/antibiotics15030322)
Supplement: Supplementary file 1 [file antibiotics-15-00322-s001.zip › antibiotics-4143767-supplementary.pdf]

A Risk Model Incorporating the Novel Inflammatory Biomarker CD64 for Predicting Bloodstream Infection in Suspected Cases

Teng Xu [1,2,†], Yu Zhou [1,2,†], Bei Wang [3], Li Wang [1,2], Yinglu Wan [1,2], Shi Wu [1,2,*] and Haihui Huang [1,2,*]

[1] Institute of Antibiotics, Huashan Hospital, Fudan University, Shanghai 200040, China; txu20@fudan.edu.cn (T.X.); 22211220091@fudan.edu.cn (Y.Z.); wanglishiny@163.com (L.W.); wan_yinglu@163.com (Y.W.)

[2] Key Laboratory of Clinical Pharmacology of Antibiotics, National Health and Family Planning Commission, Shanghai 200040, China

[3] Department of Laboratory Medicine, Huashan Hospital, Fudan University, Shanghai 200040, China; wa0261008@163.com

* Correspondence: wu_shi@fudan.edu.cn (S.W.); huanghaihui@fudan.edu.cn (H.H.); Tel.: +86-21-52888186 (S.W.); +86-21-52888190 (H.H.)

† These authors contributed equally to this work.

Supplementary Materials

Supplementary Table S1

Demographics and clinical characteristics of patients in the training set and validation set

Characteristics	Training set	Validation set	*P* value
Number of patients	185	124	
Demographics			
Age (years)	61 (48, 73)	61 (50, 72)	0.968
Male sex	67 (36)	39 (31)	0.458
Comorbidity			
Diabetes mellitus	50 (27)	26 (21)	0.281
Heart failure	4 (2)	4 (3)	0.718
Liver cirrhosis	32 (17)	22 (18)	1.000
Chronic kidney disease	14 (8)	9 (7)	1.000
Collective tissue disease	15 (8)	3 (2)	0.065
Solid tumor	30 (16)	22 (18)	0.844
Hematological malignancy	7 (4)	7 (6)	0.623
Local infection	95 (51)	63 (51)	1.000
Other underlying condition			
Corticosteroid use [a]	31 (17)	21 (17)	1.000
Immunosuppressants	14 (8)	3 (2)	0.091
Chemotherapy [a]	8 (4)	5 (4)	1.000
Invasive procedure [a]	41 (22)	34 (27)	0.355
Catheter use	90 (49)	51 ()	0.158
Laboratory analysis			
Hemoglobin (g/L)	111 (92, 132)	112 (89, 129)	0.511
White blood cell count (10^9/L)	8.1 (5.7, 11.8)	7.8 (5.5, 11.1)	0.375
Platelet count (10^9/L)	188 (117, 269)	171 (106, 252)	0.182
Alanine aminotransferase (U/L)	25 (15, 52)	34 (20, 80)	0.008
Aspartate aminotransferase (U/L)	30 (19, 64)	33 (21.75, 68.5)	0.129
Total bilirubin (μmol/L)	13.7 (9, 28.8)	13.6 (9.17, 29.3)	0.713
Direct bilirubin (μmol/L)	3.2 (1.6, 7.9)	3.8 (0.75, 7.95)	0.831
Globin (g/L)	35 (31, 38)	34 (30, 40)	0.977
Blood urea nitrogen (mmol/L)	5.7 (4.3, 8.7)	6.6 (4.9, 9.7)	0.215
Serum creatinine (μmol/L)	63 (47, 81)	60 (46, 87)	0.776

Data are presented as number (%) or median (interquartiel range) unless otherwise specified.

[a] Within 30 days before admission.

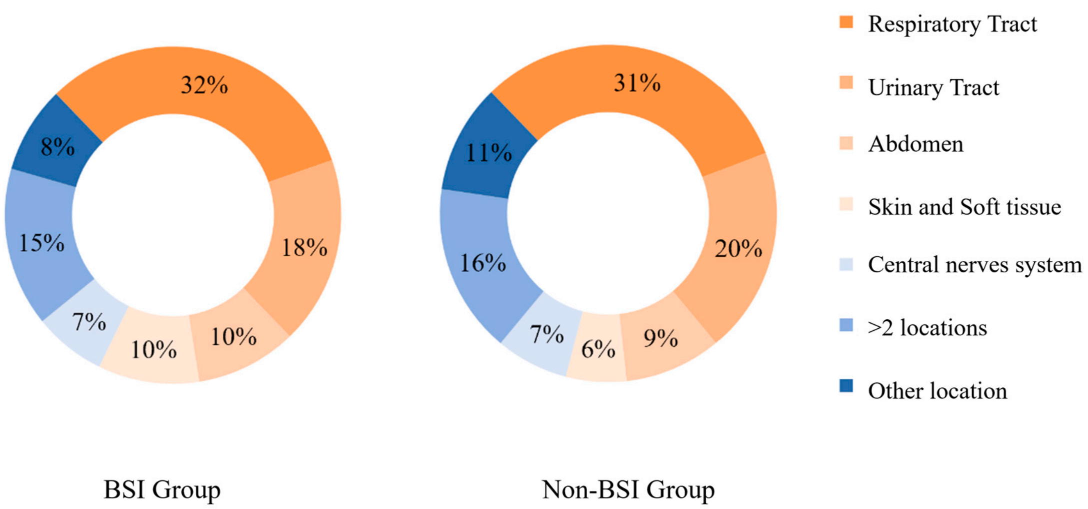

Supplementary Figure S1. Distribution of primary site of infection in the patients with bloodstream infection or without bloodstream infection. BSI, bloodstream infetion.

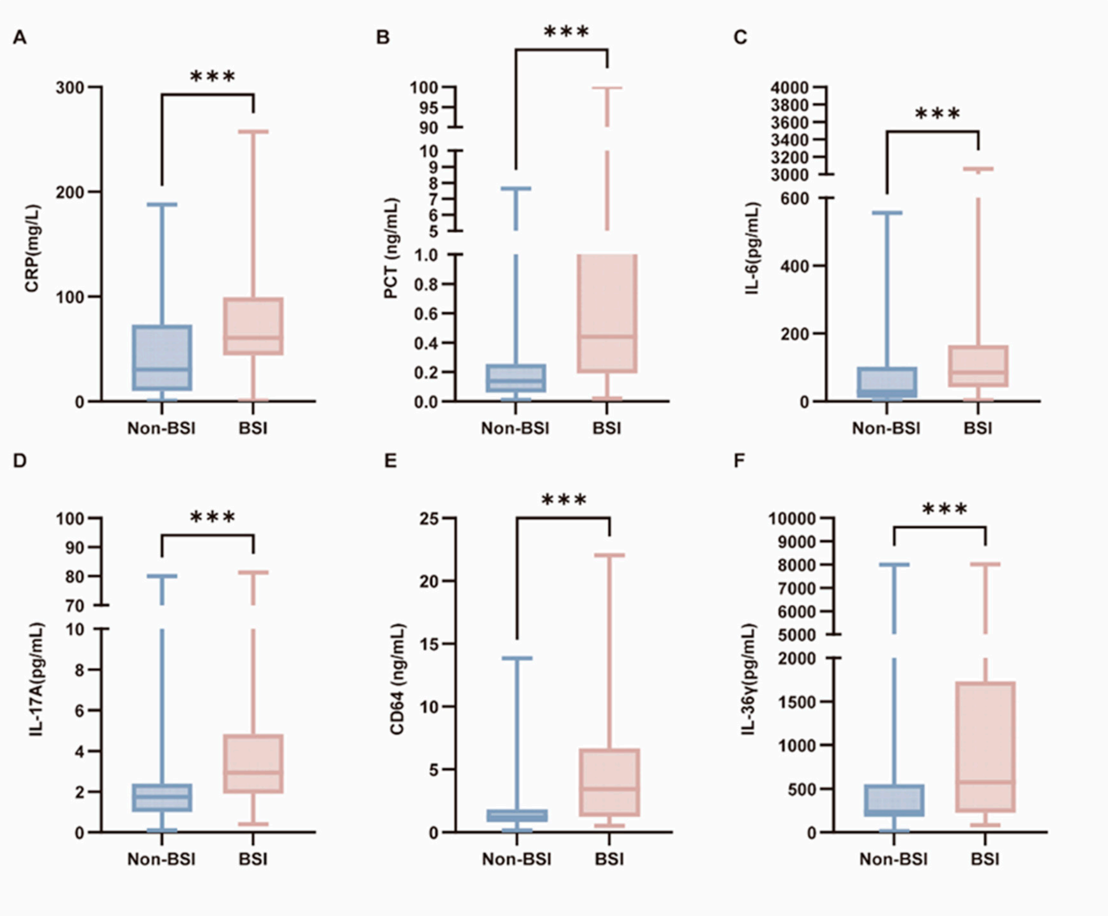

Supplementary Figure S2. Serum levels of CRP (A), PCT (B), IL-6 (C), IL-17A (D), CD64 (E), and IL-36 (F) at the time of drawing blood culture samples in patients with BSI. BSI, bloodstream infection; CRP, C-reactive protein; PCT, procalcitonin; IL, Interleukin. **P* < 0.05, ***P* < 0.01, ****P* <0.001.

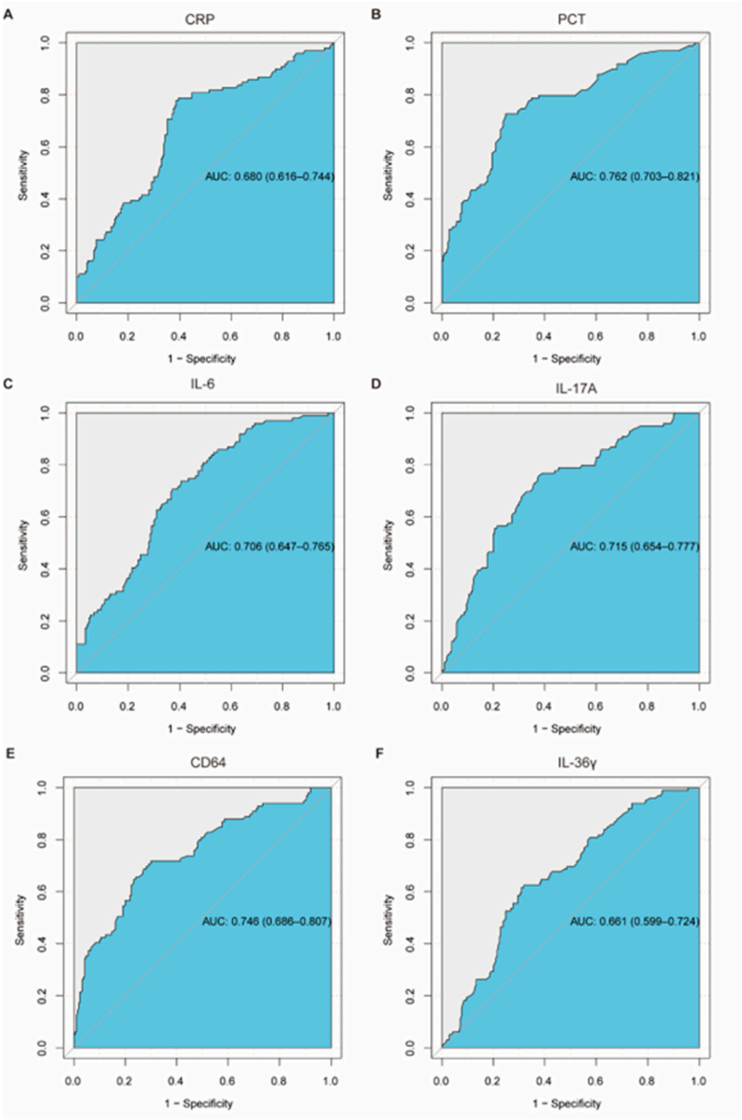

Supplementary Figure S3. The area under the receiver operating characteristic curves and the corresponding 95% CI of CRP (A), PCT (B), IL-6 (C), IL-17A (D), CD64 (E), IL-36 (F) for diagnosing bloodstream infections.

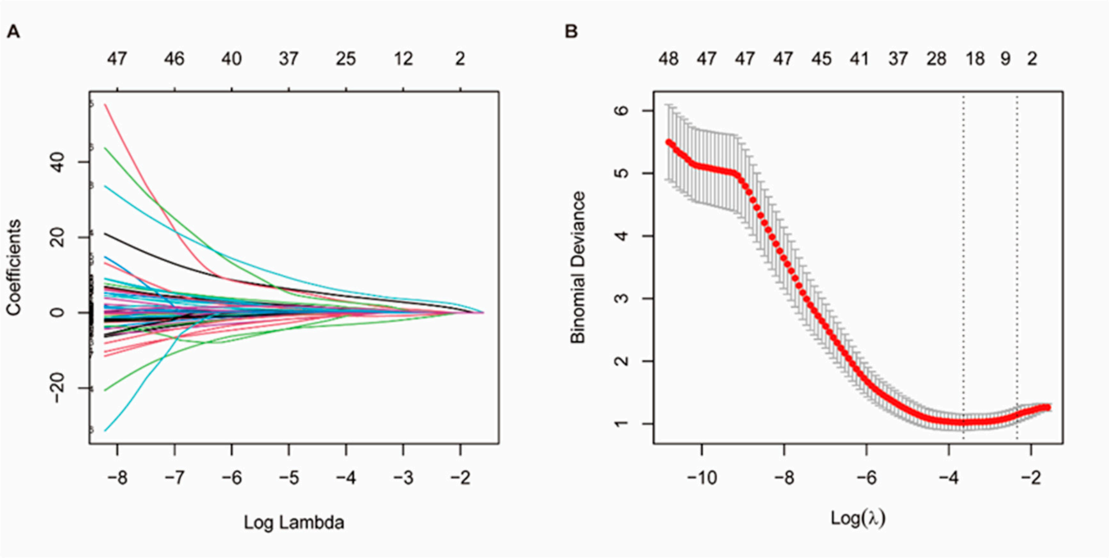

Supplementary Figure S4. Variables selection using the least absolute shrinkage and selection operator (LASSO) binary logistic regression model. (A) LASSO coefficient profiles of the candidate variables. (B) Tuning parameter (λ) selection in the LASSO model using 10-fold cross-validation via minimum criteria.
